# Supplementary material for: Simulating the structural phase transitions of metal-organic frameworks with control over the volume of nanocrystallites
Source: Commun Chem. 2023 Oct 28;6:233. doi: 10.1038/s42004-023-01025-x (PMC10613269; doi:10.1038/s42004-023-01025-x)
Supplement: Supplementary file 2 — Supplementary Information [file 42004_2023_1025_MOESM2_ESM.pdf]

Supplementary Information:

Simulating the structural phase transitions of metal-organic frameworks with control over the volume of nanocrystallites

Larissa Schaper and Rochus Schmid

1 Supplementary Note 1

Additional analysis DMOF-1

1.1 Supplementary Note 1.1

Force Constants - Applying the volume control method requires the choice of a suitable force constant for the quadratic potential. On the one hand, it is aimed to set the force constant so that the difference between the volume's mean of each NC during the simulation and the given reference volume is as small as possible to avoid substantial deviation. On the other hand, the fluctuation of the volume has to be $\Delta\Delta V \geq \pm 50$ $\mathring{A}^3$ to take snapshots from the steered MD simulation of the investigated systems every 100 $\mathring{A}^3$as initial structures for Umbrella Sampling windows.

In order to determine a force constant fulfilling the requirements, a screening experiment was performed using DMOF-1 NCs as a test system. The external potential was applied to the open pore form of the 3x3x3 NC, the 4x4x4 NC, and the 6x6x6 NC for 10 ps. The reference volume was set to the volume of a snapshot from an MD simulation in the open pore form ($V_{ref}^{3x3x3} = 9292.3$ $\mathring{A}^3$, $V_{ref}^{4x4x4} = 31483.4$ $\mathring{A}^3$, and $V_{ref}^{6x6x6} = 145199.4$ $\mathring{A}^3$).

Table 1: Supplementary Table 1: Tested force constants k for differently sized DMOF-1 NCs and the corresponding volume difference between the mean volume of the respective NC during simulation and the reference volume $\Delta V = V_{mean} - V_{ref}$ with its standard deviation.

k / $kcal \cdot mol^{-1} \cdot \AA^{-6}$	ΔV^{3x3x3} / $\AA^3$	ΔV^{4x4x4} / $\AA^3$	ΔV^{6x6x6} / $\AA^3$
0.2	-0.09±2.15	-0.1±1.7	-0.03±1.25
0.02	-1.0±8.4	-1.1±5.9	-0.1±7.1
0.002	-9.0±24.5	-10.3±18.6	-1.5±51.0
0.0002	-40.7±62.3	-76.8±50.4	-10.4±107.3
0.00002	-49.5±62.5	-221.8±97.8	-44.0±397.5
0.1	-0.2±3.5	-0.2±2.7	-0.03±2.19
0.01	-1.8±10.3	-2.1±7.2	-0.2±16.4
0.001	-13.6±31.8	-19.9±22.1	-2.4±45.9
0.0001	-45.9±60.4	-118.4±65.3	-22.4±146.3
0.00001	-44.1±67.9	-257.0±101.4	-116.8±493.4

Table 1 lists the tested force constants and the difference between the mean volume of the respective NC during the simulation and the reference volume for the respective system. In general, the mean volume of the NC is smaller than the given reference volume. The volume deviation of the same force constant differs for the differently sized MOFs, meaning a size dependency must be considered when a force constant is chosen for further calculations. The system will not adjust to the reference value if the force constant is too small, e.g., for a force constant $k = 2 \cdot 10^{-5}$ $kcal \cdot mol^{-1} \cdot \AA^{-6}$. However, smaller force constants allow, in general, a larger amplitude of the actual volume fluctuating around the reference value, which is necessary for this study to give the system a chance to reopen its pores during the simulation and to build the favored closed pore form. Based on the presented volume fluctuations, the force constant is set to 0.002 $kcal \cdot mol^{-1} \cdot \AA^{-6}$ in further SMD calculations to allow for minor differences between the mean volume and the reference volume of approximately $\Delta V = 10$ $\AA^3$ up to $\Delta V = 80$ $\AA^3$ for all investigated sizes. For this force constant the fluctuation is $\Delta\Delta V \geq \pm 50$ $\AA^3$, i.e., $\Delta\Delta V^{4x4x4} = 50.4$ $\AA^3$ up to $\Delta\Delta V^{6x6x6} = 107.3$ $\AA^3$ allowing for an appropriate choice of initial structures for follow up Umbrella Sampling calculation.

1.2 Supplementary Note 1.2

Energy conservation - The method was tested concerning energy conservation in an NVE run for 5 ps using the open pore form of the 4x4x4 NC as a test system. The reference volume corresponded to the current volume of the test system, and the force constant was set to 0.002 $kcal \cdot mol^{-1} \cdot \AA^{-6}$. The kinetic energy, the potential energy, and the total energy are shown in Figure 1. The total energy is constant during the simulation, which verifies the energy conservation of the method.

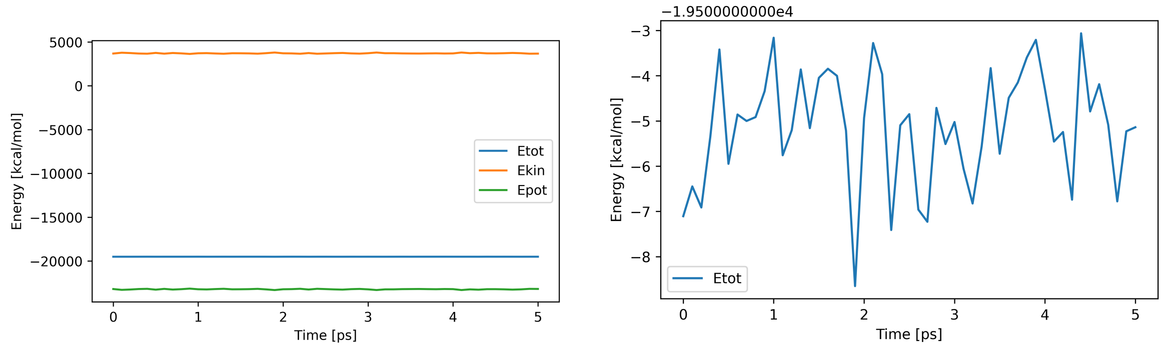

(a) The kinetic energy, the potential energy, and the total energy.

(b) Close up of the total energy.

Figure 1: Testing energy conservation using the 4x4x4 NC during a 5 ps simulation in the NVE ensemble. The total energy is constant during the simulation, which verifies the energy conservation of the method.

1.3 Supplementary Note 1.3

Umbrella sampling: Overlap of the respective Windows - To confirm that the selected windows have a sufficient overlap required for a valid free energy profile, the distributions of the collective variable (CV) were illustrated by histograms as seen in Figure 2. The overlap between adjacent histograms of the reference volume is sufficient. A more extensive overlap could be archived by additional windows or a smaller force constant allowing more volume fluctuations.

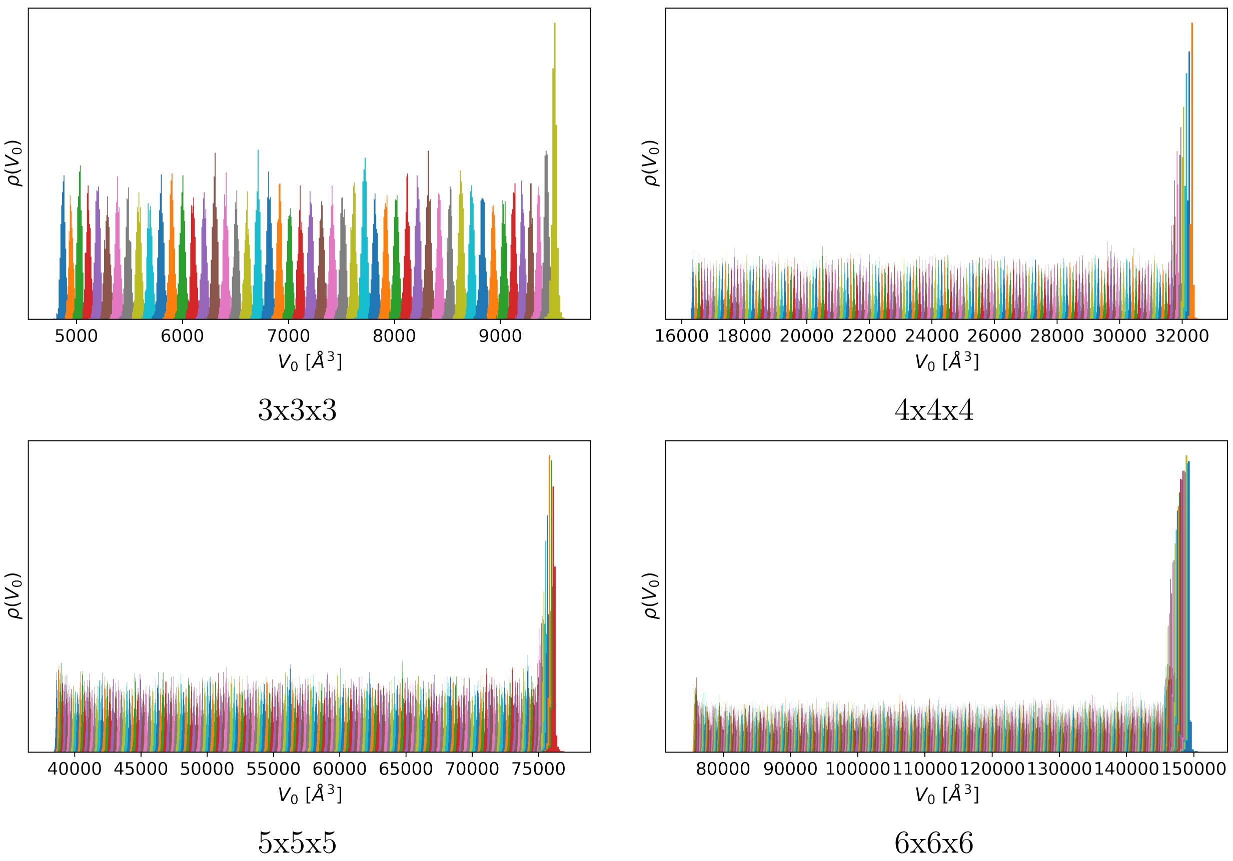

Figure 2: Probability distributions of the CV trajectories for all investigated NC of sizes. The histograms for each reference volume sufficiently overlap with the adjacent histogram.

1.4 Supplementary Note 1.4

Free energy curves normalized per number of pores - Especially for smaller NCs, it is not straightforward how the normalization of the free energy should be performed. It must be pointed out that the unnormalized total free energies of the entire NC, especially for the barriers, will determine the system's kinetic behavior as for a large molecular system. In Fig. 3, the calculated free energy curves are normalized concerning the number of pores. The overall cp to op phase barrier is substantially lower than the PBC case. The smaller systems are slightly lower in energy than the larger ones. Interestingly, the even-sized systems (4x4x4 and 6x6x6) are below the corresponding odd NCs (3x3x3 and 5x5x5).

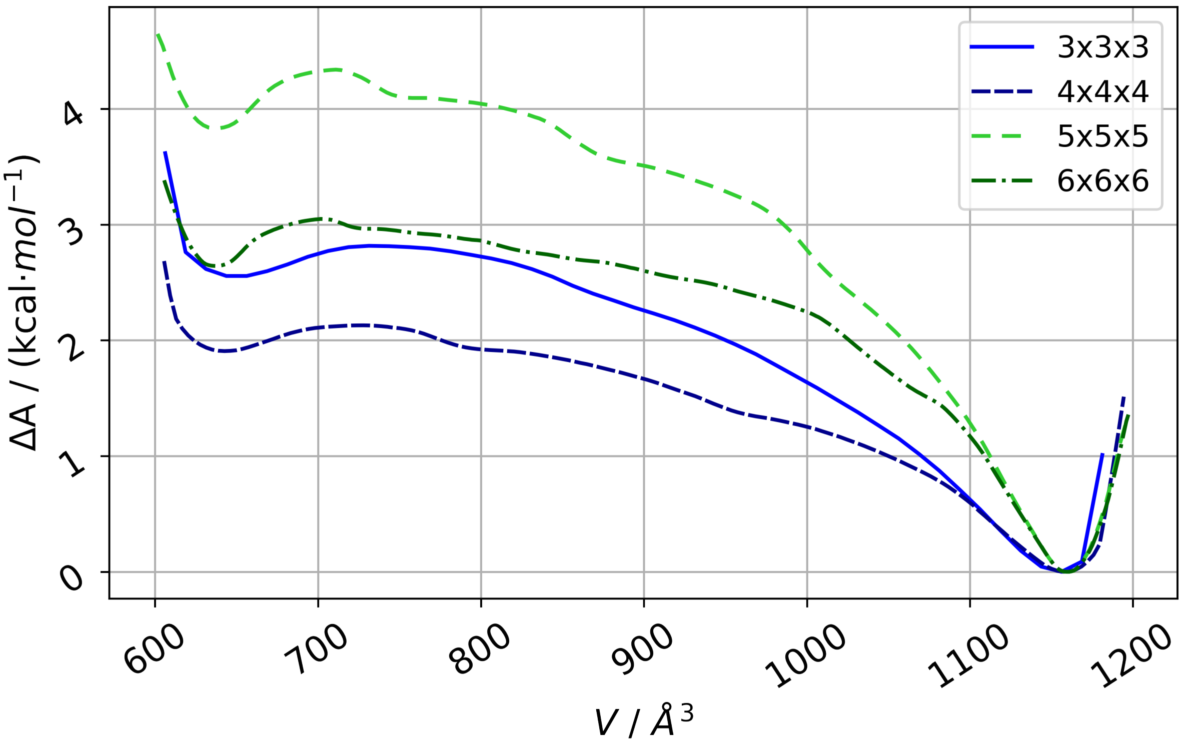

Figure 3: Free energy curves of DMOF-1 NCs normalized with respect to the number of pores.

2 Supplementary Note 2

Additional analysis DUT-128

2.1 Supplementary Note 2.1

Umbrella sampling: Overlap of the respective windows - To confirm that the selected windows have a sufficient overlap required for a valid free energy profile, the distributions of the collective variable (CV) were illustrated by histograms as seen in Figure 4. The overlap between adjacent histograms of the reference volume is sufficient. A more extensive overlap could be archived by additional windows or a smaller force constant allowing more volume fluctuations.

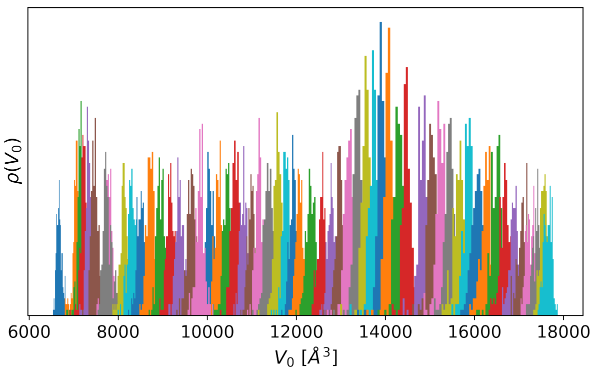

3x3x3

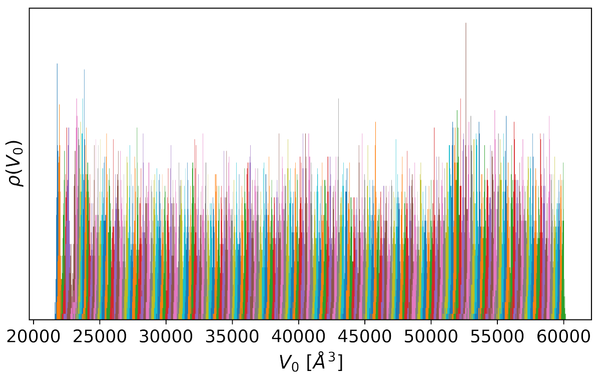

4x4x4

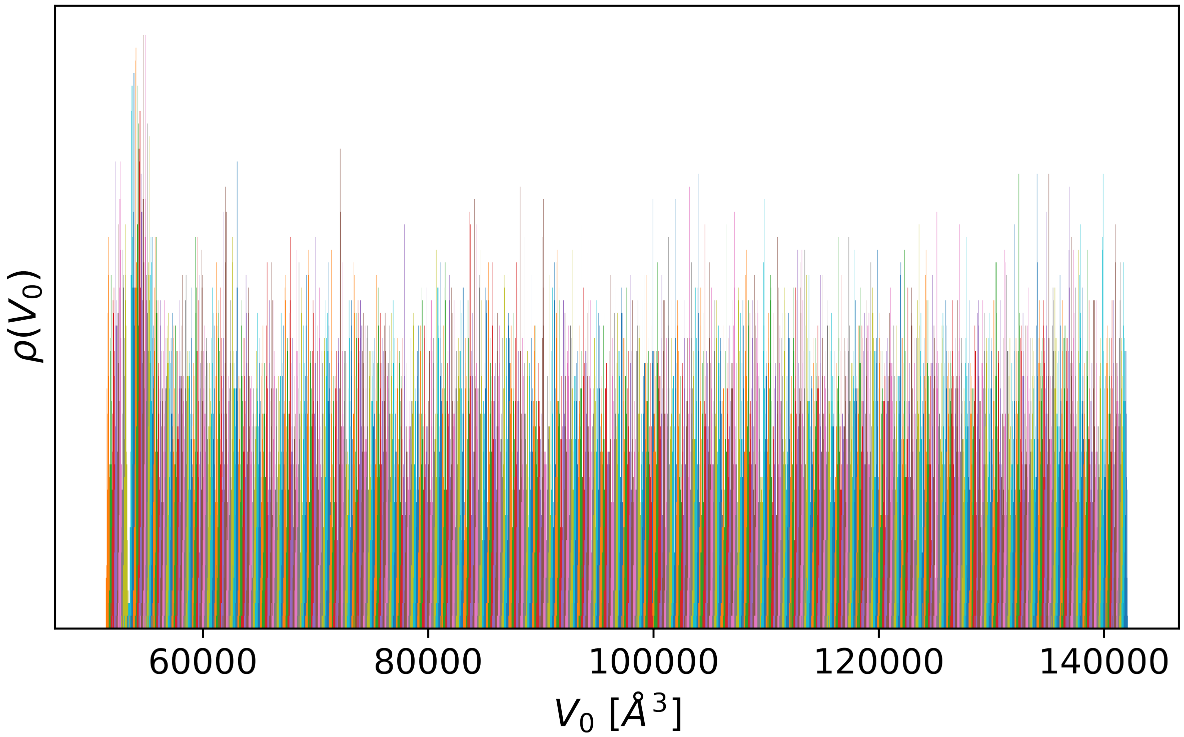

5x5x5

Figure 4: Probability distributions of the CV trajectories for all investigated NC of sizes. The histograms for each reference volume sufficiently overlap with the adjacent histogram.

2.2 Supplementary Note 2.2

Free energy curves normalized per number of pores

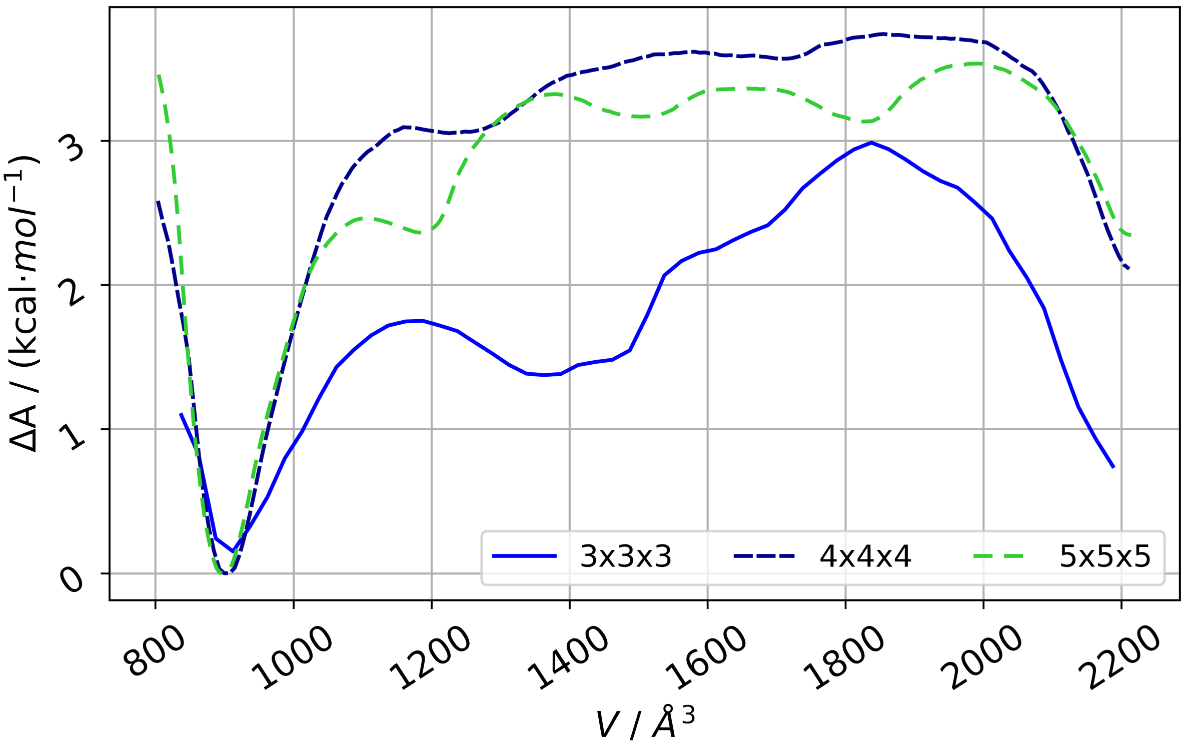

Figure 5: Free energy curves of DUT-128 NCs normalized with respect to the number of pores.
